# Associations of Total, Leisure, Occupational, and Commuting Sedentary Behavior with Overweight/Obesity Among Adults in Saudi Arabia

**DOI:** 10.3390/jcm15197670

**Published:** 2026-10-03

**Authors:** Abdullah Bandar Alansare

**Affiliations:** Department of Exercise Physiology, College of Sport Sciences and Physical Activity, King Saud University, Riyadh 11451, Saudi Arabia; aalansare@ksu.edu.sa; Tel.: +966-55-506-1381

**Keywords:** body mass index, body composition, adiposity, healthy lifestyle, sitting

## Abstract

**Objectives:** To examine the associations of total, leisure, occupational, and commuting sedentary behavior (SB) with overweight/obesity among adults in Saudi Arabia and explore whether these associations differ by SB patterns (weekdays vs. weekends) and sex (men vs. women). **Methods:** This secondary analysis of a web-based, cross-sectional survey included 948 adults living in Saudi Arabia (mean age 29.7 years; 52.1% women; 55.4% with overweight/obesity). Total and domain-specific SB were estimated using the Arabic version of the Sedentary Behavior Questionnaire. Overweight/obesity was assessed using body mass index calculated from self-reported height and weight. Adjusted logistic regression models examined the associations of total and domain-specific SB with the odds of overweight/obesity, with analyses conducted across the week and separately for week-days and weekends and stratified by sex. **Results:** Higher total and leisure SB across the week and on weekdays were associated with 3.9% to 12.3% higher odds of overweight/obesity, particularly among men (*p* < 0.05 for all). However, occupational and commuting SB were not associated with the odds of overweight/obesity (*p* > 0.05 for all). Associations were not statistically significant among women (*p* > 0.05 for all). **Conclusions:** These findings highlight the potential importance of considering the domain and temporal pattern of SB, as well as sex, when examining its associations with overweight/obesity among adults in Saudi Arabia. Further longitudinal and intervention studies are needed to establish temporal and causal relationships between the domains of SB and overweight/obesity in this population.

## 1. Introduction

Overweight and obesity represent a major and growing global public health burden. Globally, 43% of adults are estimated to be overweight and 16% to be living with obesity [1]. In the Middle East, a recent systematic review and meta-analysis reported prevalence estimates of 33.14% for overweight and 21.17% for obesity [2]. In Saudi Arabia in particular, the prevalence is similarly high, with estimates of 28.1% for overweight and 20.98% for obesity [2]. Beyond their substantial health consequences, overweight and obesity also impose considerable economic costs, accounting for approximately 2.19% of global gross domestic product (GDP) [3]. In Saudi Arabia, the economic burden is slightly higher, estimated at 2.4% of the country’s GDP [4]. These significant health and economic burdens highlight the need for effective strategies to address overweight and obesity among adults in Saudi Arabia, particularly through the promotion of healthy lifestyle modifications [5].

Sedentary behavior (SB), defined as any waking behavior characterized by an energy expenditure of ≤ 1.5 metabolic equivalents while in a seated, lying, or reclining posture [6], has emerged as an important modifiable risk factor for overweight/obesity. For instance, a previous meta-analysis including more than 100,000 adults and older adults found that higher overall SB was associated with 45% higher odds of obesity compared with lower overall SB [7]. Furthermore, each additional hour of overall SB was associated with 5.2% higher odds of overweight/obesity among Saudi men but not women [8]. Several plausible mechanisms may underlie this association, including lower energy expenditure, increased energy intake, and impaired glucose and insulin metabolism [9,10]. Prolonged, uninterrupted sitting may reduce skeletal muscle activity and glucose uptake, while SB may also coincide with unhealthy food intake, including energy-dense snacks, potentially impairing insulin sensitivity and contributing to metabolic dysfunction [9,10,11,12]. Consequently, increased energy intake during SB may promote a positive energy balance and subsequent fat accumulation. These findings suggest that SB may represent an important modifiable lifestyle target for maintaining healthy adiposity.

Of particular importance, however, SB encompasses a heterogeneous range of behaviors that may differ in their behavioral, environmental, and metabolic contexts, suggesting that its associations with overweight/obesity may vary according to the types, domains, and contexts of SB. Supporting this notion, a recent Mendelian randomization study found evidence of causal associations between genetically predicted leisure screen SB, computer use, and driving and obesity, whereas no evidence of causal association was observed for SB at work or sedentary commuting [13]. Similarly, among Saudi men, higher mentally active SB (i.e., SB involving higher cognitive demands) was associated with higher odds of overweight/obesity, while higher mentally passive SB (i.e., SB involving minimal cognitive demands) was not [8]. Collectively, these findings suggest that aggregating all SBs into a single measure may obscure potentially important differences across SB and its context. However, evidence regarding domain-specific associations of SB with overweight/obesity remains limited, particularly among adults in Saudi Arabia. Examining leisure, occupational, and commuting SB separately may therefore provide a more informative understanding of how different contexts of SB relate to overweight/obesity in this population.

Therefore, this secondary analysis examined the associations of total, leisure, occupational, and commuting SB with overweight/obesity among adults in Saudi Arabia and explored whether these associations differed by SB patterns (weekdays vs. weekends) and sex (men vs. women).

## 2. Materials and Methods

### 2.1. Study Design

The present investigation involved a secondary analysis of an existing dataset derived from a previously published study [14]. The present study was conducted according to the ethical principles of the Declaration of Helsinki. This manuscript was prepared and reported in accordance with the Strengthening the Reporting of Observational Studies in Epidemiology (STROBE) recommendations.

### 2.2. Recruitment and Data Collection

Data for the present analysis were obtained from a previously conducted web-based, cross-sectional survey examining the associations of SB with smartphone addiction among adults in Saudi Arabia [14]. The study protocol and recruitment procedures have been reported elsewhere [14]. Briefly, the survey was distributed online through official King Saud University email lists and social media platforms commonly used in Saudi Arabia, including WhatsApp, X, Snapchat, and Instagram [15]. Individuals were eligible to participate if they were adults between 18 and 65 years, living in Saudi Arabia, and were able to read the Arabic-language questionnaires. A total of 1054 participants completed the survey. Of these, 17 were excluded because of missing data or outlying values. An additional 89 participants were excluded because they were classified as underweight, as the present analyses focused on the comparison of overweight/obesity with normal weight. The final analytical sample therefore comprised 948 participants.

### 2.3. Measurements

#### 2.3.1. Participants’ Characteristics

Participant characteristics, including age, height, weight, marital status, educational status, occupational status, and chronic disease status, were self-reported (Table 1). The mean age was 29.7 years, 52.1% were women, and 55.4% had overweight/obesity. Most participants were unmarried (63.4%), while 49.4% were currently students and 42.2% were employed. The majority of them did not report a diagnosed chronic disease (88.8%). The median durations of total, leisure, occupational, and commuting SB were 8.9, 6.0, 0.7, and 1.0 h/day, respectively.

#### 2.3.2. Overweight/Obesity

BMI was derived from participants’ self-reported height and weight using the following formula: BMI (kg/m^2^) = weight (kg)/height (m)^2^. BMI was then classified into 4 categories: (1) underweight (BMI < 18.5 kg/m^2^), (2) normal weight (BMI between 18.5 and 24.9 kg/m^2^), (3) overweight (BMI between 25 and 29.9 kg/m^2^), and (4) obese (BMI ≥ 30 kg/m^2^). For the present analyses, the underweight group was omitted, and the overweight and obesity categories were collapsed into one overweight/obesity group. The normal weight group was retained as the reference category for the comparisons of the odds of overweight/obesity.

#### 2.3.3. Total and Domain-Specific Sedentary Behavior

Total, leisure, occupational, and commuting SB across the week, on weekdays, and on weekends were estimated using the Arabic version of the Sedentary Behavior Questionnaire (SBQ) [16,17]. This SBQ assessed 9 types of SB, comprising 7 leisure, 1 occupational, and 1 commuting SB. The leisure SB category consisted of 7 types of SB, including watching TV or videos on a smartphone or tablet while seated, playing electronic and video games while seated, sitting and reading a printed book or magazine or reading from a smartphone or tablet, doing artwork or crafts, sitting and listening to an audio recording on a radio, smartphone, or tablet, playing a musical instrument, and sitting and talking on the phone. The occupational SB category consisted of time spent sitting while doing paperwork or computer work (e.g., sending emails or paying bills). In contrast, the commuting SB category consisted of sitting and driving or riding a car, bus, or train. Total SB was calculated by summing time spent across all 9 SB types. For each SB category, average daily SB across the week was calculated using the following formula: [(weekday hours × 5) + (weekend hours × 2)]/7.

### 2.4. Statistical Analysis

Participants’ characteristics and total and domain-specific SB levels were summarized using descriptive statistics. Continuous variables were summarized using means ± standard deviations (SDs) or medians and interquartile ranges (IQRs), as appropriate. Categorical variables were summarized using frequencies and percentages. To examine the associations of total SB with overweight/obesity, logistic regression models were fitted with total SB entered as the predictor, adjusting for age, sex, marital status, education, occupation, and chronic disease status. To evaluate the associations of domain-specific SB with overweight/obesity, logistic regression models were fitted with leisure, occupational, and commuting SB entered simultaneously as the predictors, adjusting for age, sex, marital status, education, occupation, and chronic disease status. These analyses were repeated with weekdays and weekends entered simultaneously into the same model and were then stratified by sex (men and women). The statistical analyses were performed using JASP (version 0.95.4.0, JASP Team, University of Amsterdam, Amsterdam, Netherlands). The significance level was set at *p* < 0.05.

## 3. Results

Table 2 displays the associations of total and domain-specific SB across the week with overweight/obesity in adults. Each additional hour of total and leisure SB across the week was associated with 3.9% and 5.6% higher odds, respectively, of overweight/obesity (*p* < 0.05 for both). Sex-stratified analyses revealed that these associations were stronger and limited to men, among whom each additional hour of total and leisure SB across the week was associated with 8.0% and 8.3% higher odds, respectively, of overweight/obesity (*p* < 0.05 for both). No significant associations were observed for occupational or commuting SB (*p* > 0.05 for all).

When the analyses were repeated for weekdays and weekends separately, the associations of total and leisure SB were evident only on weekdays (Table 3). Specifically, among the overall sample, each additional hour of leisure SB on weekdays was associated with 6.5% higher odds of overweight/obesity (*p* < 0.05). Among men, each additional hour of total and leisure SB on weekdays was associated with 11.6% and 12.3% higher odds, respectively, of overweight/obesity (*p* < 0.05 for both). No other significant associations were observed across SB domains and patterns, or among women (*p* > 0.05 for all).

## 4. Discussion

This study simultaneously examined the associations of total, leisure, occupational, and commuting SB with overweight/obesity among adults in Saudi Arabia and explored whether these associations varied by SB patterns and sex. Higher total and leisure SB, particularly on weekdays, were associated with higher odds of overweight/obesity, whereas occupational and commuting SB were not. Notably, these associations were only evident among men. These findings suggest that considering the domain and pattern of SB, as well as sex-specific associations, may provide greater insight into the relationship between SB and overweight/obesity than relying solely on total SB. Further longitudinal and intervention studies are needed to confirm these findings, clarify the underlying mechanisms, and inform targeted SB reduction strategies to prevent overweight/obesity.

A previous systematic review and meta-analysis reported that adults and older adults with higher overall SB had 45% higher odds of obesity than those with lower overall SB [7]. However, the included studies were conducted in populations from North and South America, Europe, Australia, and Africa, leaving uncertainty about whether similar findings apply to Middle Eastern populations, particularly in Saudi Arabia. Addressing this gap, the present study found that each additional hour/day of total SB was associated with 3.9% higher odds of overweight/obesity among adults living in Saudi Arabia. Although the direction of the association was consistent with previous findings [7], the magnitude of the association should not be directly compared due to important methodological differences between studies. The present study estimated the change in odds per additional hour/day of SB, whereas the previous systematic review and meta-analysis primarily compared adults with higher vs. lower SB levels. Moreover, differences in SB assessment methods (self-report vs. self-report alongside device-based measures) and adiposity outcomes (overweight/obesity vs. obesity alone) may have contributed to the difference in the magnitude of the observed associations [7]. Population-specific behavioral and lifestyle characteristics may have further contributed to the observed discrepancies [18,19]. These findings extend the existing evidence by demonstrating a positive association between total SB and overweight/obesity among adults living in Saudi Arabia and suggest that SB may represent a potentially modifiable behavioral target.

The present study further suggests that leisure SB may be an important contributor to the observed association of total SB with overweight/obesity. Specifically, each additional hour/day of leisure SB was associated with 8% higher odds of overweight/obesity, whereas occupational and commuting SB were not associated. These findings are partially consistent with results from the China Health and Nutrition Survey, which reported that higher leisure SB was associated with 6.5% to 12.2% higher odds of excessive weight among Chinese adults and older adults [20]. Likewise, a previous systematic review examining occupational SB found inconsistent evidence across cross-sectional studies and no prospective evidence supporting a causal relationship between higher occupational SB and BMI [21]. One possible explanation is that leisure SB often involves prolonged, uninterrupted sitting and may be accompanied by unhealthy snacking and higher exposure to food advertising [22]. In contrast, occupational SB often occurs within a structured work routine that may involve interruptions by standing and walking for meetings or other work-related tasks, potentially resulting in less prolonged uninterrupted sitting than leisure SB [23]. Thus, the stronger association observed for leisure SB may reflect differences in the behavioral context and accompanying lifestyle behaviors across SB domains.

Contrary to the present findings indicating that commuting SB was not associated with overweight/obesity, previous studies have reported that individuals who primarily use passive modes of transportation (i.e., accumulating greater commuting SB) have a higher risk of obesity than those who engage in active commuting [24,25,26]. This discrepancy may partly reflect differences in how commuting behavior was assessed. Previous studies generally compared passive and active commuting modes [24,25,26], thereby capturing differences in both SB and PA. Conversely, the present study specifically examined whether each additional hour/day of commuting SB was associated with overweight/obesity, therefore isolating the sedentary component. The null association observed in the present study may consequently not be directly comparable with findings based on commuting mode. Nonetheless, this explanation remains speculative, and future robust studies are needed to clarify the independent contribution of commuting SB to overweight/obesity.

Importantly, the associations of total and leisure SB with overweight/obesity were detected only on weekdays and only among men. These pattern- and sex-specific associations may reflect differences in the behavioral contexts in which SB occurs, sex-related differences in the accumulation and composition of SB, or residual confounding by unmeasured factors. The weekday-specific associations may partly reflect the more structured and habitual SB during the working weekdays, whereas SB on weekends is often more variable [27,28,29]. Similarly, the sex-specific associations may reflect differences in the composition of leisure SB, with men engaging in more leisure screen-based SB and women spending more time sitting and reading [30,31]. In addition, although several important covariates were adjusted for, potentially relevant factors, such as PA, sleep, and diet, were not measured and may have contributed to the observed differences. A better understanding of when, where, and how different types of SB are accumulated may help explain why associations with adiposity vary across SB domains, temporal patterns, and sex groups.

### 4.1. Strengths and Limitations

The present study has notable strengths. First, it included a relatively large sample of adults living in Saudi Arabia, which enhanced statistical power and facilitated sex-stratified analyses [32]. Second, SB was assessed across multiple domains, including leisure, occupational, and commuting SB. Furthermore, the assessment of leisure SB captured a broad range of SBs, including screen-based and non-screen-based SB and mentally active and passive SB, providing a more detailed characterization of leisure SB.

Several limitations should be considered when interpreting the findings. First, occupational and commuting SB were each assessed with a single SBQ item, whereas leisure SB was assessed with seven items, which may have resulted in differences in measurement precision across SB domains [33]. Second, the use of a self-report SB questionnaire may have introduced recall and reporting bias and may have underestimated SB compared with device-based measures [33,34]. Third, the web-based, cross-sectional design increases the potential for self-selection and reporting biases and precludes causal inference [35]. Finally, although the analyses adjusted for several relevant covariates, including age and socioeconomic factors, residual confounding cannot be excluded because other potentially important variables, such as PA [36], were not measured and therefore could not be included in the models. Future longitudinal studies and randomized trials should combine validated self-report questionnaires with objective devices to more comprehensively characterize SB across leisure, occupational, and commuting domains while accounting for important potential confounders.

### 4.2. Clinical Implications

Current global lifestyle recommendations for the prevention and management of overweight/obesity emphasize a combination of healthy dietary patterns, regular PA, and other health-related behaviors, including adequate sleep and reducing SB [5]. Given that growing evidence suggests that SB domains may not be equally associated with overweight/obesity [20,21,24,25,26], the present findings suggest that SB reduction strategies may benefit from considering the domain and context of SBs rather than treating all SBs as equivalent, particularly among adults in Saudi Arabia. Specifically, leisure SB on weekdays may warrant greater attention as a potentially relevant target for future SB reduction interventions. However, because of the cross-sectional design, these findings should not be interpreted as evidence that reducing leisure or total SB would directly prevent or reduce overweight/obesity. Longitudinal and intervention studies are required to determine whether targeting specific domains of SB, particularly leisure SB, can contribute to the prevention and management of overweight/obesity.

## 5. Conclusions

In sum, higher total and leisure SB were associated with higher odds of overweight/obesity, particularly on weekdays and among men in Saudi Arabia. Conversely, higher occupational and commuting SB were not associated. These findings highlight the potential importance of considering the domain and temporal pattern of SB, as well as sex, when examining its associations with overweight/obesity. Further robust studies are needed to establish temporal and causal relationships between SB domains and overweight/obesity, clarify why associations may differ across SB domains and contexts and between men and women, and determine whether reducing specific domains of SB could contribute to comprehensive strategies for the prevention or management of overweight/obesity among adults in Saudi Arabia.

## Figures and Tables

**Table 1:** Characteristics and Total and Domain-Specific SB Levels of the Included Participants (*n* = 948).

Characteristic	Mean ± SD, Median [IQR], or *n* (%)
Age (years old)	29.7 ± 11.6
Weight (kg)	72.9 ± 17.6
Height (cm)	165.6 ± 9.3
BMI (kg/m^2^)	26.4 ± 5.5
Normal Weight	423 (44.6)
Overweight/Obese	525 (55.4)
Sex	
Men	454 (47.9)
Women	494 (52.1)
Marital Status	
Married	347 (36.6)
Unmarried	601 (63.4)
Education	
Diploma Degree or Less	288 (30.4)
Undergraduate Degree	455 (48.0)
Graduate Degree	205 (21.6)
Occupational Status	
Student	468 (49.4)
Employed	400 (42.2)
Currently Unemployed	57 (6.0)
Retired	23 (2.4)
Diagnosed Chronic Disease	
Yes	106 (11.2)
No	842 (88.8)
Total SB (h/day)	
Across the Week	8.9 [5.9]
On Weekdays	9.1 [6.5]
On Weekends	8.0 [6.0]
Leisure SB (h/day)	
Across the Week	6.0 [4.6]
On Weekdays	6.3 [4.5]
On Weekends	5.8 [5.0]
Occupational SB (h/day)	
Across the Week	0.7 [2.5]
On Weekdays	1.0 [2.8]
On Weekends	0.3 [1.0]
Commuting SB (h/day)	
Across the Week	1.0 [1.5]
On Weekdays	1.0 [1.5]
On Weekends	1.0 [1.5]

BMI: body mass index; cm: centimeter; IQR: interquartile range; kg: kilogram; m^2^: meter squared; *n*: number; SB: sedentary behavior; SD: standard deviation.

**Table 2:** Associations of Total, Leisure, Occupational, and Commuting SB across the Week with Overweight/Obesity in Adults (*n* = 948).

Variable	SB Across the Week (h/day)			
	Total *	Leisure #	Occupational #	Commuting #
	OR (95% CI)			
Overall Sample (*n* = 948)				
Normal Weight	Reference			
Overweight/Obesity	**1.039**	**1.056**	1.001	0.982
	**(1.008, 1.071)**	**(1.017, 1.097)**	(0.911, 1.099)	(0.868, 1.111)
Men (*n* = 454) ∞				
Normal Weight	Reference			
Overweight/Obesity	**1.080**	**1.083**	1.143	1.007
	**(1.030, 1.133)**	**(1.018, 1.152)**	(0.969, 1.347)	(0.839, 1.208)
Women (*n* = 494) ∞				
Normal Weight	Reference			
Overweight/Obesity	1.018	1.039	0.941	0.985
	(0.977, 1.060)	(0.988, 1.092)	(0.833, 1.061)	(0.826, 1.175)

CI: confidence interval; OR: odd ratio; SB: sedentary behaviour. Bold indicates significant associations (*p* < 0.05). * indicates models were adjusted for age, sex, marital status, education, occupation, and chronic disease status. # indicates models were adjusted for age, sex, marital status, education, occupation, and chronic disease status, and simultaneously adjusted for the other SB. ∞ indicates models were adjusted for age, marital status, education, occupation, and chronic disease status, and simultaneously adjusted for the other SB.

**Table 3:** Associations of Total, Leisure, Occupational, and Commuting SB on Weekdays and Weekends with Overweight/Obesity in Adults (*n* = 948).

Variable	SB on Weekdays (h/day)				SB on Weekends (h/day)			
	Total *	Leisure #	Occupational #	Commuting #	Total *	Leisure #	Occupational #	Commuting #
	OR (95% CI)							
Overall Sample (*n* = 948)								
Normal Weight	Reference							
Overweight/Obesity	1.034	**1.065**	0.994	0.917	1.004	0.990	1.017	1.079
	(0.990, 1.080)	**(1.007, 1.124)**	(0.904, 1.094)	(0.776, 1.083)	(0.962, 1.048)	(0.938, 1.044)	(0.902, 1.147)	(0.923, 1.261)
Men (*n* = 454) ∞								
Normal Weight	Reference							
Overweight/Obesity	**1.116**	**1.123**	1.145	1.014	0.959	0.958	0.968	0.993
	**(1.037, 1.200)**	**(1.027, 1.228)**	(0.973, 1.347)	(0.798, 1.288)	(0.890, 1.034)	(0.877, 1.047)	(0.777, 1.205)	(0.797, 1.237)
Women (*n* = 494) ∞								
Normal Weight	Reference							
Overweight/Obesity	0.995	1.027	0.945	0.853	1.024	1.008	1.013	1.169
	(0.940, 1.054)	(0.956, 1.104)	(0.833, 1.072)	(0.666, 1.092)	(0.970, 1.081)	(0.941, 1.080)	(0.871, 1.178)	(0.927, 1.474)

CI: confidence interval; OR: odd ratio; SB: sedentary behaviour. Bold indicates significant associations (*p* < 0.05). * indicates models were adjusted for age, sex, marital status, education, occupation, and chronic disease status, and simultaneously adjusted for total SB on weekdays and weekends. # indicates models were adjusted for age, sex, marital status, education, occupation, and chronic disease status, and simultaneously adjusted for the other SB. ∞ indicates models were adjusted for age, marital status, education, occupation, and chronic disease status, and simultaneously adjusted for the other SB.

## Data Availability

The data are available upon request from the corresponding author.

## Abbreviations

The following abbreviations are used in this manuscript:

BMI	Body Mass Index
CI	Confidence interval
cm	Centimeter
IQR	Inter Quartile Range
GPD	Global Gross Domestic Product
kg	Kilogram
m^2^	Meter Squared
n	Number
OR	Odds Ratio
SB	Sedentary Behavior
SBQ	Sedentary Behavior Questionnaire
SD	Standard Deviation
STROBE	Strengthening the Reporting of Observational Studies in Epidemiology
